# Supplementary material for: Effects of Three Lipidated Oxytocin Analogs on Behavioral Deficits in CD38 Knockout Mice
Source: Brain Sci. 2017 Oct 16;7(10):132. doi: 10.3390/brainsci7100132 (PMC5664059; doi:10.3390/brainsci7100132)
Supplement: Supplementary File 1 [file brainsci-07-00132-s001.pdf]

Supplementary table 1. Summary of parental scores data.

	mean ± SEM	n	P value
WT dam run 1	9.5 ± 1.1	10	0.07
WT dam run 2	10.0 ± 0	10	> 0.1
WT dam run 3	10.0 ± 0	10	> 0.1
$CD38^{-/-}$ dam run 1	6.0 ± 1.4	10	0.07
$CD38^{-/-}$ dam run 2	7.6 ± 1.3	10	> 0.1
$CD38^{-/-}$ dam run 3	8.2 ± 0.9	10	> 0.1
WT sire run 1	6.1 ± 0.9	10	< 0.01
WT sire run 2	6.6 ± 1.0	10	< 0.01
WT sire run 3	7.1 ± 1.2	10	< 0.01
$CD38^{-/-}$ sire run 1	0.9 ± 0.1	10	< 0.01
$CD38^{-/-}$ sire run 2	1.1 ± 0.5	10	< 0.01
$CD38^{-/-}$ sire run 3	0.6 ± 0.3	10	<0 .01

Supplementary table 2. Summary of parental scores data. P values compared with $CD38^{-/-}$ treated by PBS

	mean ± SEM	n	P value
	**30 minutes after injection**		
WT + PBS	8.2 ± 0.75	13	0.001
$CD38^{-/-}$ + PBS	1.0 ± 0.53	13	
$CD38^{-/-}$ + OT	5.4 ± 1.3	8	0.0158
$CD38^{-/-}$ + LOT-1	4.1 ± 1.6	8	0.247
$CD38^{-/-}$ + LOT-2	8.9 ± 0.48	8	0.001
$CD38^{-/-}$ + LOT-3	8.4 ± 0.56	8	0.001
	24 hours after injection		
$CD38^{-/-}$ + OT	1.6 ± 1.2	8	0.999
$CD38^{-/-}$ + LOT-1	6.5 ± 1.5	8	0.015
$CD38^{-/-}$ + LOT-2	4.3 ± 1.6	8	0.515
$CD38^{-/-}$ + LOT-3	3.0 ± 1.5	8	0.999

Supplementary table 3A. Rapid test of social acquisition and recognition. The time spent for social investigation in each trial for 1 min

	mean ± SEM 1 trial	mean ± SEM 2 trial	mean ± SEM 3 trial	mean ± SEM 4 trial	mean ± SEM 5 trial	n
WT PBS	49 ± 1.59	50 ± 1.12	45 ± 1.54	38 ± 1.66	50 ± 0.99	10
WT OT	49 ± 1.20	47 ± 2.93	43 ± 2.47	38 ± 1.99	48 ± 1.21	10
$CD38^{-/-}$ PBS	41 ± 1.67	43 ± 1.83	39 ± 2.7	36 ± 1.71	32 ± 1.85	15
$CD38^{-/-}$ OT	40 ± 2.49	36 ± 1.16	35 ± 2.58	29 ± 1.29	39 ± 1.63	5
$CD38^{-/-}$ LOT-1	36 ± 3.72	36 ± 2.93	31 ± 2.84	27 ± 1.43	36 ± 3.26	6
$CD38^{-/-}$ LOT-2	39 ± 3.31	34 ± 4.18	31 ± 1.77	32 ± 3.15	46± 4	5
$CD38^{-/-}$ LOT-3	44 ± 2.47	36 ± 2.68	37 ± 5.2	28 ± 1.93	42 ± 6.04	5

Supplementary table 3B. The percent changes are illustrated between the 1st and 4th trials. P value compared with $CD38^{-/-}$ treated by PBS

	mean ± SEM	n	P value
WT PBS	22.16 ± 2.06	10	> 0.9999
WT OT	22.14 ± 2.55	10	> 0.9999
$CD38^{-/-}$ PBS	19.62 ± 3.7	15	
$CD38^{-/-}$ OT	32.88 ± 6.8	5	> 0.9999
$CD38^{-/-}$ LOT-1	18.96 ± 10.6	6	> 0.9999
$CD38^{-/-}$ LOT-2	18.58 ± 4.35	5	> 0.9999
$CD38^{-/-}$ LOT-3	45.14 ± 7.5	5	0.0197

Supplementary table 3C. The percent changes are illustrated between the 4th and 5th trials. P value compared with $CD38^{-/-}$ treated with PBS.

	mean ± SEM	n	P value
WT PBS	23.74 ± 3.11	10	< 0.0001
WT OT	21.81 ± 2.85	10	0.0011
$CD38^{-/-}$ PBS	-5.09 ± 3.69	15	
$CD38^{-/-}$ OT	32.45 ±5.69	5	0.0337
$CD38^{-/-}$ LOT-1	20.44 ± 9.39	6	> 0.9999
$CD38^{-/-}$ LOT-2	28.74 ± 9.69	5	0.0434
$CD38^{-/-}$ LOT-3	34.55 ± 16.5	5	0.3752

Supplementary table 4. Tail suspension test. The immobility time by male and female. P value compared with CD38-/- treated with PBS.

	**mean ± SEM**	**n**	**P value**
		**Males**	
WT + PBS	144.3 ± 9.95	15	0.0008
WT + OT	158.8 ± 7.2	5	
CD38-/- + PBS	76.4 ± 9.12	18	0.009
$CD38^{-/-}$ + OT	152.8 ± 25.12	5	0.0211
$CD38^{-/-}$ + LOT-1	88.2 ± 18.24	6	> 0.9999
$CD38^{-/-}$ + LOT-2	144.6 ± 26.28	6	0.0330
$CD38^{-/-}$ + LOT-3	135.1 ± 19.48	6	0.1234
		**Females**	
WT + PBS	169.1 ± 8.65	10	< 0.0001
WT + OT	166.5 ± 33.92	5	0.0011
$CD38^{-/-}$ + PBS	53.7 ± 10.27	10	
$CD38^{-/-}$ + OT	137.7 ± 33.51	5	0.0337
$CD38^{-/-}$ + LOT-1	75.4 ± 18.35	5	> 0.9999
$CD38^{-/-}$ + LOT-2	135.5 ± 15.54	5	0.0434
$CD38^{-/-}$ + LOT-3	114.9 ± 14.37	5	0.3752

Supplementary table 5. Elevated plus maze test. The time in open arms and distance travelled in open arms. P value compared with CD38-/- treated with PBS.

	mean ± SEM	n	P value
	**Time travelled in open arms**		
WT + PBS	42.77 ± 10.93	10	> 0.9999
WT + OT	75.45 ± 20.73	4	0.3008
CD38$^{-/-}$ + PBS	27.5 ± 9.1	5	
CD38$^{-/-}$ + OT	52.08 ± 15.58	6	> 0.9999
	**Distance travelled in open arms**		
WT + PBS	0.94 ± 0.26	10	> 0.9999
WT + OT	1.06 ± 0.3	4	> 0.9999
CD38$^{-/-}$ + PBS	0.66 ± 0.25	5	
CD38$^{-/-}$ + OT	1.29 ± 0.43	6	> 0.9999

Supplementary table 6. Sucrose preference test. The preference for 1% sucrose solution consumption. P value compared with CD38-/- treated with PBS

	mean ± SEM	n	P value
	**Females**		
WT + PBS	0.67 ± 0.04	13	> 0.9999
WT + OT	0.63 ± 0.05	6	> 0.9999
CD38$^{-/-}$ + PBS	0.72 ± 0.05	20	
CD38$^{-/-}$ + OT	0.72 ± 0.04	5	> 0.9999
	**Males**		
WT + PBS	0.78 ± 0.04	10	< 0.0001
WT + OT	0.77 ± 0.03	10	< 0.0001
CD38$^{-/-}$ + PBS	0.46 ± 0.04	7	
CD38$^{-/-}$ + OT	0.68 ± 0.05	7	0.0068
	**Males 30 min after injection**		
CD38$^{-/-}$ + PBS	0.46 ± 0.04	7	
CD38$^{-/-}$ + OT	0.69 ± 0.05	7	0.0128
CD38$^{-/-}$ + LOT-1	0.58 ± 0.05	5	> 0.9999
CD38$^{-/-}$ + LOT-2	0.69 ± 0.05	5	0.0293
CD38$^{-/-}$ + LOT-3	0.53 ± 0.04	5	> 0.9999
	**Females 24 hours after injection**		
CD38$^{-/-}$ + PBS	0.46 ± 0.04	7	
CD38$^{-/-}$ + OT	0.43 ± 0.04	5	> 0.9999
CD38$^{-/-}$ + LOT-1	0.69 ± 0.06	5	0.0304
CD38$^{-/-}$ + LOT-2	0.53 ± 0.06	5	> 0.9999
CD38$^{-/-}$ + LOT-3	0.43 ± 0.04	5	> 0.9999

Supplementary table 7. Locomotor activity in mouse pups. P value compared with CD38-/- treated with PBS

	mean ± SEM	n	P value
WT + PBS	3.7 ± 0.71	25	0.0001
CD38$^{-/-}$ + PBS	7.5 ± 0.3	25	
CD38$^{-/-}$ + OT	4.2 ± 0.6	10	0.0811
CD38$^{-/-}$ + LOT-1	5.3 ± 0.86	10	> 0.9999
CD38$^{-/-}$ + LOT-2	2.9 ± 0.8	10	0.0009
CD38$^{-/-}$ + LOT-3	2.5 ± 0.78	10	0.0002
